# Superficial siderosis due to filum terminale tumor: An uncommon cause of cranial nerve dysfunction

**DOI:** 10.1002/ccr3.1645

**Published:** 2018-12-05

**Authors:** Tom Campion, Farrukh Arfeen, Ashok Adams

**Affiliations:** ^1^ Imaging Department Barts Health NHS Trust London UK

**Keywords:** Haemosiderosis, sensorineural hearing loss

## Abstract

If superficial siderosis is suspected based on clinical presentation, susceptibility weighted imaging should be undertaken in addition to standard MRI sequences as it is more sensitive than T2 weighted imaging. Once diagnosed, imaging of the entire brain and spine must be undertaken to assess for an underlying cause.

## 1 INTRODUCTION

Superficial siderosis is a potential cause of sensorineural hearing loss, and the development of more sensitive MRI techniques, specifically susceptibility weighted imaging, has made it easier to detect. We present a case of a woman with sensorineural hearing loss and difficulty swallowing due to superficial siderosis secondary to a spinal myxopapillary ependymoma, and highlight the importance of an imaging strategy that enabled the identification of this uncommon but important diagnosis.

## 2 CASE HISTORY

A previously healthy 46‐year‐old woman presented with difficulty swallowing. She also reported headache, vertigo, loss of balance, and hearing loss. On examination, she had left sided sensorineural hearing loss, multidirectional rotational gaze‐evoked nystagmus, and impaired tandem gait. A barium swallow study and CT head examination were both normal.

**Figure 1:**
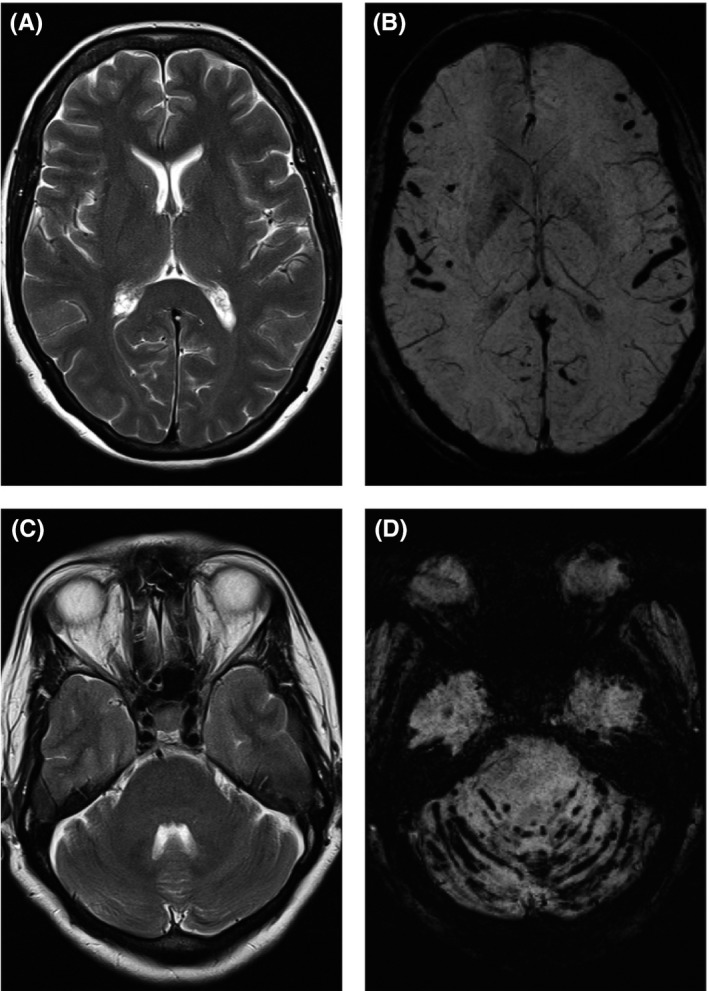
MRI head. Selected axial T2 weighted images of the supratentorial (A) and infratentorial (C) compartment that appear normal. However, the corresponding axial susceptibility weighted images (B and D) demonstrate extensive supratentorial and infratentorial blooming artifact reflecting sulcal pial hemosiderosis

An MRI head was performed given the multiple neurological deficits. The susceptibility weighted images demonstrated extensive supratentorial and infratentorial hemosiderosis (Figure 1). The other routine T1 and T2 weighted sequences were normal and intracranial time‐of‐flight MR angiography did not reveal any source of bleeding.

**Figure 2:**
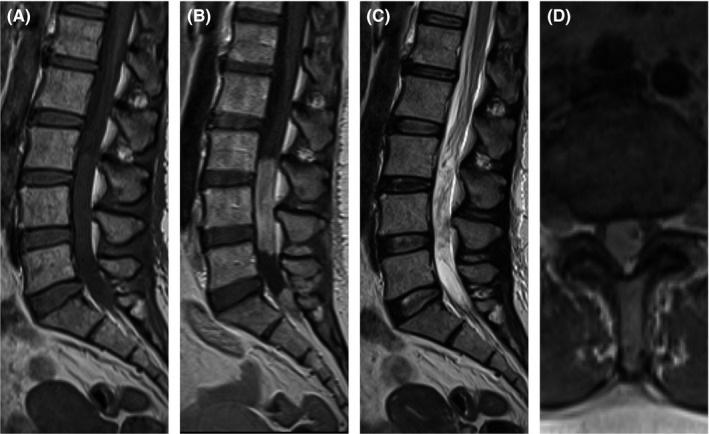
MRI spine, selected images. Sagittal T1 pre‐(A) and post‐(B) contrast, T2 weighted (C) and axial T1 postcontrast (D) images demonstrate T1‐isointense, T2‐hyperintense intradural and extramedullary lesion centered on the filum terminale which enhances avidly following contrast administration. Radiological appearances are compatible with a myxopapillary ependymoma

The patient was referred to neurology, and further investigated with an MRI of the whole spine to determine if there was an occult source of bleeding. This revealed an intradural enhancing lesion centered on the filum terminale that was deemed the likely source of the bleeding (Figure 2). The lesion was confirmed as a myxopapillary ependymoma (WHO grade 1) following surgical resection. She had spinal radiotherapy for residual nodules, but the majority of her symptoms resolved apart from the sensorineural hearing loss. Follow‐up MRI was stable; the changes associated with the hemosiderosis did not resolve but did not progress.

## 3 DISCUSSION

With the increasing availability of more advanced blood‐sensitive sequences, the diagnosis of superficial siderosis is being made more frequently. The constellation of symptoms was described in the 1990s with sensorineural hearing loss, cerebellar ataxia, and pyramidal signs the most commonly recognized signs.^1^ The condition results from the deposition of hemosiderin in the subpial layers of the central nervous system.^2^ Initially this begins with the abnormal presence of red blood cells that contain heme which is neurotoxic. Ferritin is produced by glial cells (microglia and Bergmann glia) to bind the heme into hemosiderin; however, the production of ferritin is eventually overwhelmed with resultant neuronal damage.^3^

Superficial siderosis is subclassified into “infratentorial” superficial siderosis given its predilection for the posterior fossa and to distinguish it from the cortical superficial siderosis associated with cerebral amyloid angiopathy or previous isolated subarachnoid hemorrhage. The posterior fossa predominance is thought to be due to the presence of large numbers of Bergmann glia (with high levels of ferritin synthesis) in the cerebellum as well as the flow of CSF over this area. The vestibulocochlear nerve is also often affected due to its long glial segment and abundant microglia compared to other cranial nerves, hence the prevalence of sensorineural hearing loss (as seen in our case).^3^, ^4^

The classical imaging features of superficial siderosis are T2 hypointensity associated with blooming artifact on T2* weighted imaging along the cerebellar, cerebral, and spinal surfaces as well as the intrathecal spinal nerve roots and cisternal portions of the cranial nerves.^3^ There may later be signs of neuronal damage such as gliosis and atrophy.

A recent review of 65 cases of infratentorial superficial siderosis reported that there were no cases in which blooming artifact on gradient echo/SWI sequences was not associated with concurrent T2 hypointensity.^4^ This is also highlighted by multiple previous case studies.^5^, ^6^ In our case, the T2 weighted imaging was normal and only the susceptibility weighted imaging (SWI) was able to demonstrate the extensive siderosis highlighting the importance of using blood‐sensitive sequences.

Susceptibility weighted imaging is a relatively new MRI technique, which incorporates both magnitude and, crucially, phase information. Phase information is important because it is more sensitive to local changes in magnetic fields, due to substances with a different susceptibility (magnetic response to an external magnetic field) such as blood or iron.^7^ This offers additional information over T2* or gradient‐echo sequences and increases the sensitivity and the resolution.

Once the diagnosis of superficial siderosis has been established, an underlying cause must be sought. In a group of 48 infratentorial superficial siderosis patients with a classical triad of symptoms and a chronic and progressive history, 27% had a cause demonstrated on brain MRI, 52% on spinal MRI, and 10% on CT myelography (although CT myelography was only performed on 10% of the cohort). None had additional potentially causative findings demonstrated on angiography. Dural abnormalities were the most common cause, for example, due to previous traumatic or iatrogenic injuries. Only one had a spinal tumor as seen in our case. Other causes included pineal tumors, a thrombosed giant aneurysm, and a thalamostriate tumor.^4^

This highlights the importance of initial complete brain and spine MR imaging in the investigation of these cases; this was particularly true in our case, as the myxopapillary ependymoma seen was clinically silent. Despite the small number of spinal tumors seen in the largest case series, there have been a number of case reports of myxopapillary ependymomas causing superficial siderosis.^5^, ^6^, ^8^, ^9^ Of note, SWI has not been previously reported in these cases (to our knowledge), and in only one other case was the siderosis not detectable on T2 imaging, as in our case.^8^ With removal of the tumor, the majority of symptoms resolved with the exception of sensorineural hearing loss, which remained stable consistent with previous cases.^5^ The persistence of the hearing loss is thought to be due to the particular susceptibility of the vestibulocochlear nerve to hemosiderosis and likely resulting early damage to the nerve itself.^10^

## 4 CONCLUSION

Superficial siderosis is an important diagnosis and may be missed without appropriate imaging—we believe SWI is the most sensitive imaging sequence for its detection. As this case demonstrates, the cause of the superficial siderosis may be remote and clinically silent and as such imaging of the entire brain and spine is required.

## ETHICAL STANDARDS

The authors assert that all procedures contributing to this work comply with the ethical standards of the relevant national and institutional guidelines on human experimentation (NHS Health Research Authority & Research Ethics Committee) and with the Helsinki Declaration of 1975, as revised in 2008. Informed consent was obtained from the patient discussed in this report.

## CONFLICT OF INTEREST

None declared.

## AUTHOR CONTRIBUTION

TC: performed literature search and manuscript preparation. FA: performed manuscript editing and figure preparation. AA: performed case identification, manuscript editing; as principle investigator, takes overall responsibility for the integrity of the content of the manuscript.
